# Root-Knot-Nematode-Encoded CEPs Increase Nitrogen Assimilation

**DOI:** 10.3390/life13102020

**Published:** 2023-10-07

**Authors:** Shova Mishra, Weiming Hu, Peter DiGennaro

**Affiliations:** Entomology and Nematology Department, University of Florida, Gainesville, FL 32611, USA; shovamishra@ufl.edu (S.M.); huweiming2009@gmail.com (W.H.)

**Keywords:** CEP, plant-peptide, root-knot nematode, nitrogen uptake, isotope labeling

## Abstract

C-terminally encoded peptides (CEPs) are plant developmental signals that regulate growth and adaptive responses to nitrogen stress conditions. These small signal peptides are common to all vascular plants, and intriguingly have been characterized in some plant parasitic nematodes. Here, we sought to discover the breadth of root-knot nematode (RKN)-encoded CEP-like peptides and define the potential roles of these signals in the plant–nematode interaction, focusing on peptide activity altering plant root phenotypes and nitrogen uptake and assimilation. A comprehensive bioinformatic screen identified 61 CEP-like sequences encoded within the genomes of six root-knot nematode (RKN; *Meloidogyne* spp.) species. Exogenous application of an RKN CEP-like peptide altered *A. thaliana* and *M. truncatula* root phenotypes including reduced lateral root number in *M. truncatula* and inhibited primary root length in *A. thaliana*. To define the role of RKN CEP-like peptides, we applied exogenous RKN CEP and demonstrated increases in plant nitrogen uptake through the upregulation of nitrate transporter gene expression in roots and increased 15N/14N in nematode-formed root galls. Further, we also identified enhanced nematode metabolic processes following CEP application. These results support a model of parasite-induced changes in host metabolism and inform endogenous pathways to regulate plant nitrogen assimilation.

## 1. Introduction

Root-knot nematodes (RKNs) feed on plant roots as obligatory sedentary parasites that induce and maintain an intimate relationship with their host. The demonstrative phenotype of this interaction is the development of highly metabolically active and novel root cell types near the vasculature called ‘giant cells’ [1]. Such feeding sites presumably serve as the primary nutrient source for nematode growth, development, and completion of their life cycle [2]. The formation of these atypical plant cell types and structures that serve as feeding sites likely involves the secretion of nematode proteins or effectors [3]. The most relevant nematode secretions to this work are those similar to endogenous plant peptide signals in terms of sequence and function [4,5]. Endogenous plant signal peptides regulate a wide array of developmental processes [6] and plant–pathogen interactions. One well-described family is that of C-terminally Encoded Peptide (CEP), which were first characterized in *A. thaliana* and are well described throughout vascular plants [7,8,9]. Interestingly, CEP-like peptides were also identified in a giant cell forming sedentary endoparasitic nematode, *Meloidogyne* spp., and syncytia forming semi-endo-parasite, *Rotylenchulus reniformis* [4,10]. The precise role of nematode-encoded CEP-like peptides is still not fully understood, but their presence offers a valuable tool and resource for gaining a deeper comprehension of plant peptide signaling and the development of nematode feeding sites.

In plants, endogenous CEP regulates plant development, including root and shoot growth, lateral root, and nodule formation in legumes [11,12,13,14]. Plant peptides frequently exhibit post-translational modifications that may play roles in peptide activity, storage, transport, among other functions [15,16,17,18], with CEP displaying hydroxyprolination. Plant-encoded CEPs are also linked to nitrogen and carbon status [13,19] and respond to nitrogen stress conditions by upregulating nitrate transporter genes [20,21], indicating functionally diverse roles for different CEP sequences in plant physiology. One likely scenario is that *CEP* genes are upregulated in response to nitrogen starvation [19,21] and that the active peptides move in the xylem to the leaves where they initiate a signal cascade that results in the upregulation of nitrate transporter genes in the roots [21,22,23], indicating a role for CEP in nitrate uptake. However, in *Arabidospsis*, primary root growth is limited when there is an upregulation of nitrate transporter genes [11], probably as an adaptive response to the limited nitrogen condition. Further expanding the roles of this family of peptides, in *M. truncatula,* CEPs form circumferential root swelling [12,13] when applied exogenously, and this swelling is due to cortical cell hyperplasia, phenotypically similar to the root galls induced by root-knot nematodes. Additionally, another role of CEP in plant nutrient uptake has been functionally characterized using stable isotope labeling [24], where exogenous MtCEP1D1:hyp4,11 and AtCEP1:hyp4,11 enhanced nitrate, phosphate, and sulphate uptake in both model plants *M. truncatula* and *A. thaliana* [24].

These signal peptides common to all vascular plants are also encoded in the genome of plant-parasitic nematode, *Meloidogyne hapla* [4], some of which exhibit significant sequence similarity to a plant, *M. truncatula*, a CEP that plays a role in nodule formation [12,13]. Many similarities exist between root-knot-nematode-induced galls and rhizobia-induced nodules [25,26,27]. With an expansive host range, *M. hapla* presumably communicates multiple CEP signals across various hosts, such that nematode CEP mimics native plant CEP to facilitate the establishment of its parasitic relationship. A large family of CEP genes are also encoded in another plant-parasitic nematode, *Rotylenchulus reniformis.* One member of the *R. reniformis* CEP is involved in maintaining parasitism as shown by upregulation of the CEP transcripts during host–nematode interaction [10]. The *R. reniformis* CEP is also involved in nitrogen signaling in plants, indicating a role for CEPs in regulating plant biology. However, the impact in increasing nitrogen transporter expression on the plant–nematode interaction is still unknown. Peptide mimicry by nematodes to regulate feeding-cell differentiation during parasitism is already demonstrated with other signal peptide families [28]. One plausible and well-supported theory suggests a role for nematode CEP to upregulate the expression of nitrate transporter genes and plant nutrient uptake [21,24].

Here, we screened the available genomes of *Meloidogyne* species for the presence of putative CEP-like peptides and characterized nematode-encoded CEP involvement in nematode nitrogen uptake using stable isotope labeling. Exploring the role of nematode-encoded CEP in plant physiology and nematode parasitism will reveal novel insights into plant peptide signaling metabolism and biotic interactions.

## 2. Materials and Methods

### 2.1. Screening Putative CEPs in Meloidogyne Species

Members of the CEP family were identified within the genomes of seven different RKN species currently available. A database was built using the 15-amino-acid mature region from known *A. thaliana* and *M. truncatula* CEPs and were used as a query. To identify CEP-like peptides, all RKN genomes were scanned for open reading frames (ORFs) between 30 and 150 AA long using the program getorf-EMBOSS package [29]. SignalP3.0 was used to search each ORF for secretion signal sequences. ORF that lacked a signal sequence and sequences that contain >5 cysteine residues were excluded from analysis to limit cysteine-rich proteins and non-secreted products. A database of *A. thaliana* and *M. truncatula* was used as query for a BLASTP search in the reduced ORF of the RKN species. Putative CEPs within each RKN species were numbered in the order they are encoded within the reference genome.

### 2.2. Logo Plot and Phylogenetic Analyses

To depict any potential sequence similarity, a sequence logo plot of RKN *A. thaliana-* and *M. truncatula*-conserved CEP domains, of about 15 amino acids, was built using webLogo (https://weblogo.berkeley.edu/logo.cgi, accessed on 8 september 2005). Briefly, a sequence logo is created from a collection of aligned sequences and depicts the consensus sequence and diversity of the sequences. To depict sequence similarities of individual sequences, a phylogenetic tree was generated using the maximum likelihood method and JTT-matrix-based model [30]. The peptide sequences from RKN and plants were first aligned using MUSCLE and a tree was generated from 100 rounds of bootstrapping in Mega-X [31]. *A. thaliana* CLE peptides were used as an outgroup.

### 2.3. Root Architecture Analysis

To observe if nematode CEP induces similar gross plant morphological phenotypes, bioassays were conducted through exogenous application of peptides on *A. thaliana* and *M. truncatula* seedlings. MtCEP1D1:hyp4,11 (AFQ{HYP}TTPGNS{HYP}GVGH), MhCEP11:hyp4,11 (AFR{HYP}TAPGHS{HYP}GVGH), and AtCEP1:hyp4,11 (DFR{HYP}TNPGNS{HYP}GVGH) peptides were chemically synthesized at 95% purity by GenScript USA Inc., where {HYP} indicates hydroxyproline. *A. thaliana* were grown in Murashige and Skoog (MS) media for one week and transferred to another Petri plate containing MS medium with or without peptides. *M. truncatula* were grown in fahraeus medium with no nitrogen supplemented. Where indicated, synthetic peptides corresponding to AtCEP1:hyp4,11, MtCEP1D1:hyp4,11, and MhCEP11:hyp4,11 were added to the medium before solidification at a final concentration of 1 µM. One-week-old *M. truncatula* seedlings were transferred to 9 cm plastic Petri plates (www.Simport.com) containing CEP and plants were held vertically in a growth chamber at 24 °C with a 16 h day length. For *A. thaliana* and *M. truncatula*, each treatment had 16 and 8 biological replicates, respectively. Two weeks after transferring seedlings to peptide-treated plates, seedlings were imaged using a calibrated color optical scanner (STD4800). Using the image, primary root length was measured using ImageJ software and number of lateral roots was counted. Root length and lateral root numbers were subjected to Statistical Analysis System (SAS) using Proc Glimmix and means were separated using Tukey’s test.

### 2.4. Nitrate Transporter Analysis

Endogenous plant CEPs upregulate the transcription of a nitrate transporter gene [20,21]; therefore, we assayed the ability of the nematode CEP-like domain to alter nitrate transporter gene expression (*NRT2.1*) in *M. truncatula*. Peptides for MhCEP11:hyp4,11 and MtCEP1D1:hyp4,11 were chosen for this study as MhCEP11:hyp4,11 is most sequentially similar to MtCEP1D1:hyp4,11 [4]. *M. truncatula* seeds were surface-sterilized, soaked in water for 24 h to break dormancy, and sown into 9 cm diameter Petri plates containing fahraeus medium with no nitrogen [32]. One-week-old *M. truncatula* seedlings were transferred to another 9 cm diameter Petri plate containing fahraeus medium with or without synthesized peptides corresponding to MhCEP11:hyp4,11 and MtCEP1D1:hyp4,11. Where indicated, synthetic peptides corresponding to MtCEP1D1:hyp4,11-hyP and MhCEP11:hyp4,11 were added to the medium before pouring to achieve the desired concentration of 1 µM. Each treatment was repeated independently three times and each replicate included whole roots from two plants. Plants were held vertically in a growth chamber at 24 °C with a 16 h day length and roots were collected at 7 d, 15 d, and 21 d post treatment in peptides. Roots were flash-frozen and stored at −80 °C until required.

Total RNA was isolated from samples collected at 7 d, 15 d, and 21 d post treatment in peptide media from roots of *M. truncatula* seedlings using Trizol reagent according to manufacturer’s instructions. cDNA was synthesized using the iscript Reverse Transcription Supermix (BioRAD Laboratories, USA) for RT-qPCR according to manufacturer’s protocols. RT-qPCR was carried out using a StepOne Plus Real Time PCR detection system (Applied Biosystem, USA) using SYBR Green as detection agent. PCR reactions were conducted with three technical replications for each sample in MicroAmp Fast 96-well reaction plate (0.1 mL) (Applied Biosystems, USA). The total volume of the reaction was 10 μL with 0.5 μL of forward and reverse primers each at 10 μM, 2.5 μL of cDNA template, 1.5 μL of nuclease-free water, and 5 μL of SYBR Green qPCR MasterMix (iTaq Universal SYBR Green Supermix, Bio-RAD Laboratories, USA). PCR was performed at 95 °C for 20 s followed by 40 cycles at 95 °C for 3 s and 60 °C for 30 s, and the melting curve was generated at 95 °C for 15 s, 60 °C for 1 min, and 95 °C for 15 s. Ct value was calculated using the StepOne software V2.3 (Applied Biosystems, USA).

The mean Ct value for each biological replicate was calculated in Excel from three PCR technical replicates. Relative expression level of *NRT2.1* gene was determined by calculating fold change (2^−ΔΔCt^) [33] using *M. truncatula* ubiquitin gene (UBQ10; TC100142) [34] as a housekeeping gene for normalization. Data obtained after fold change calculation were analyzed using Student’s *t*-test.

### 2.5. Nitrogen Uptake Analysis in Nematode Using Mass Spectrometry

To elucidate if MhCEP11:hyp4,11 increases nematode nitrogen uptake and assimilation, we employed stable isotope labeling coupled with high-throughput mass spectrometry. Plants were labeled using the rare and stable nitrogen isotope 15N by substituting KNO_3_ within Hoagland’s solution with KN^15^O_3_, generating a modified Hoagland’s solution. Three-week-old *M. truncatula* seedlings were supplied with 15 mL of 0.4X modified Hoagland’s solution that contained 15 mM of 15 N nitrogen, three times, every other day, over the course of 1 week. *M. truncatula* were treated with 1 uM of MhCEP11:hyp4,11 and were compared to untreated control (water) plants; treatments occurred 1 day after the third application of 15 N enriched fertilizer. Treated plants received MhCEP11:hyp4,11 for two consecutive days at same 1 uM concentration. Two days post-peptide treatment, all plants were inoculated with 1000 J^2^ of *M. hapla*. To quantify nitrogen uptake by nematodes, root gall samples were collected at 21 days post inoculation. Extracted nematode galls, including accompanying root tissues, were ground in a mortar and pestle with liquid N_2_ into a lysate. Lysate containing nematode and plant protein were sent to UF-ICBR proteomics and Mass Spectrometry Core for protein analysis. Briefly, isolated proteins, after quality assessment, were digested with trypsin and 2 µg of protein was used for mass spectrometry (MS). Mass spectrometry followed the standard protocol to measure mass-to-charge ratio of molecules. After running MS, Proteome Discover 2.4 (PD 2.4) software was used to determine proteins and peptide hits. Proteins were identified using a >95% false discovery rate (FDR) and peptides were identified whenever the peptide spectrum match (PSM) confidence was at least high. Resultant peptides were then mapped to the nematode protein database in uniprot. A dataset of labeled nematode proteins was generated individually for three biological replicates and the abundance ratio (15N/14N) for each protein was calculated in all samples using PD 2.4.

The mean abundance ratio (15N/14N) of proteins was calculated for each sample. Mean abundance ratios were analyzed using Student’s *t*-test where comparisons were conducted between samples treated with MhCEP11:hyp4,11 and with no peptide control. A list of all labeled proteins from both treatments was assigned to known Gene Ontology (GO) enrichment analysis to predict their overrepresented functional roles and associated biological processes using g:Profiler website [35]. 

## 3. Results

### 3.1. Meloidogyne Genomes Encode Suites of CEP-like Peptides

**Figure 1:**
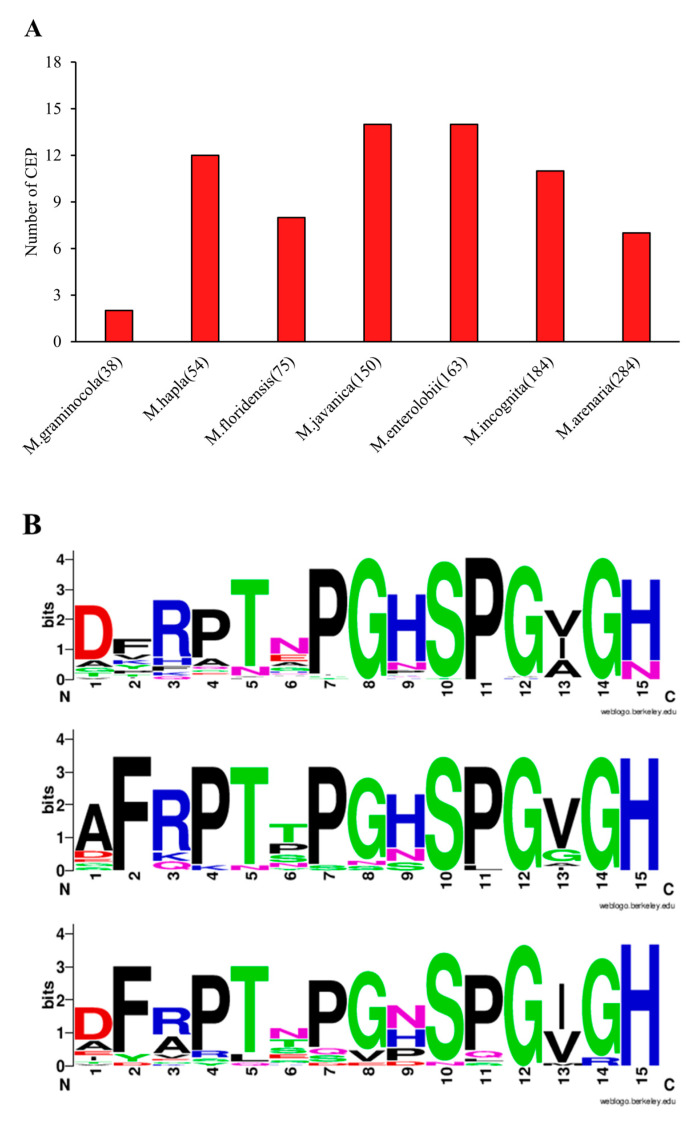
C-terminally encoded peptides in RKN species and CEP ligand domains of RKN and plants. (**A**) Number of CEPs discovered in different genomes of RKN. *X*-axis indicates different RKN species with their genome size (Mb) included in parentheses. Y-axis indicates the number of the CEPs identified in RKN genomes. (**B**) Sequence logo generated for Meloidogyne spp. (top), *M. truncatula* (middle), and *A. thaliana* (bottom). The relative sizes of the letters indicate their frequency in the sequences.

**Figure 2:**
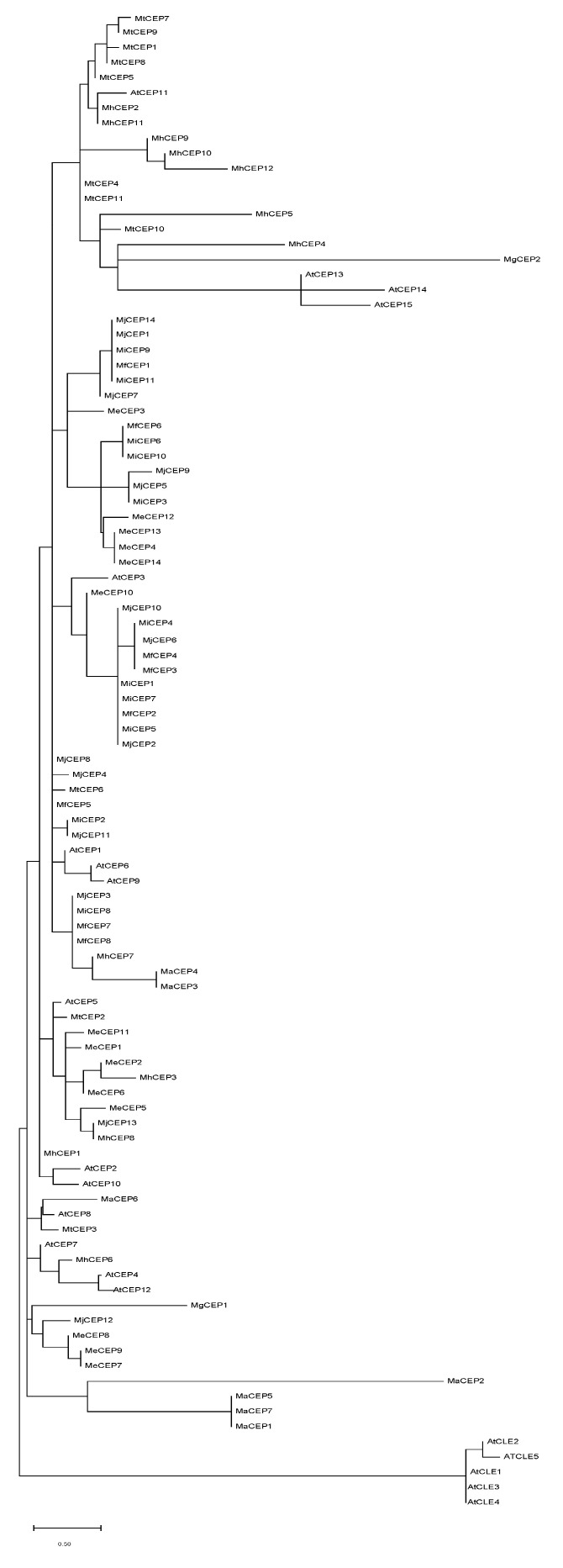
Phylogenetic tree of CEPs of *Meloidogyne*, *M. truncatula*, and *A. thaliana*. Conserved CEP domains were grouped into functional clades based on primary sequence information. The tree is drawn by using maximum likelihood and JTT-matrix-based model method with 100 bootstrap replicates. Bootstrap values are not significant except for the outgroup.

Genomes of seven RKN species were screened for CEP-like peptides using *M. truncatula* and *A. thaliana* conserved CEP domains as a query. Among all RKN species examined, we identified conserved CEP-like domains consisting of 12–15 amino acids. In total, we discovered 61 CEP-like peptides across the RKN species. The number of CEPs varied among RKN species ranging from 2 to 14 regardless of the genome size as depicted in Figure 1A. In each RKN species, CEPs were named with numbers in the order they appear in the genome. Root-knot nematode CEP-like peptides are presented in Table S1. Logo plots were constructed to observe the similarity between RKN and plant CEPs and show that RKN CEPs look similar to *M. truncatula* and *A. thaliana* CEPs with 13 out of 15 matching residues (Figure 1B). Phylogenetic analyses did not show distinct clades delineating CEP domains by species, with a bootstrap support of less than 0.4%. There were no clear patterns of clustering that would correspond to specific species. Instead, CEP sequences from various RKN species exhibited random clustering with plant CEP, except for the outgroup (Figure 2). These findings indicate that the phylogenetic relationships among CEP domains are more complex than anticipated, with cross-species similarities and random clustering observed.

### 3.2. Role of CEP in Plant Root Architecture

**Figure 3:**
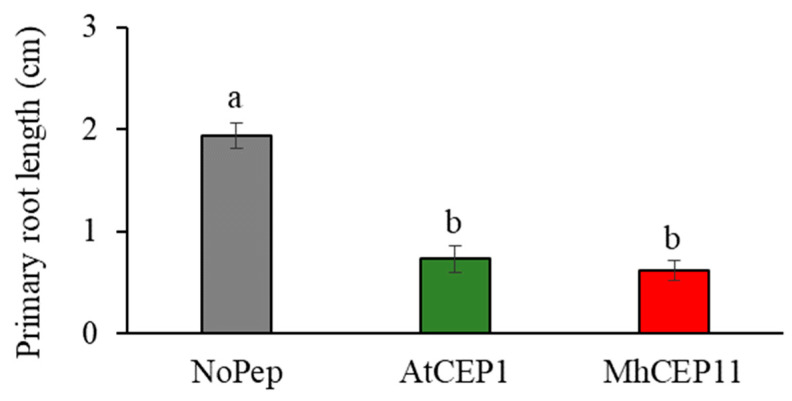
Primary root length in *A. thaliana* two weeks post imbibition with peptides. Bars on the column represent standard error and bars with different letters are significantly different (*p* < 0.05). Data shown include 16 biological replications.

**Figure 4:**
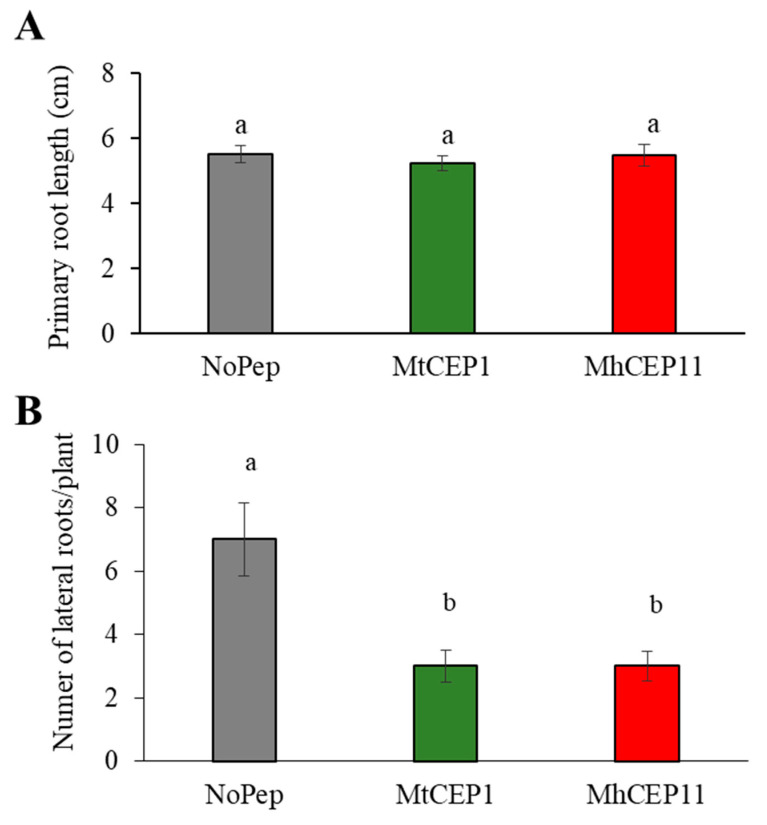
Root architecture measurement in *M. truncatula* two weeks post imbibition with peptides. Root length (**A**) and number of lateral roots (**B**) were measured for any effect of peptides. Bars on the column represent standard error and bars with different letters are significantly different (*p* < 0.05). Data shown include 8 biological replications.

Plant CEPs affect plant root architecture in a number of ways [11]. To determine if nematode CEPs can impart similar plant phenotypes, we assayed the effect of exogenous application of MhCEP11:hyp4,11 in two different hosts, *A. thaliana* and *M. truncatula*. For *A. thaliana,* we used AtCEP1:hyp4,11, and for *M. truncatula,* we used MtCEP1D1:hyp4,11 as a positive control as they have already shown root phenotypes in previous studies [9,13]. In both hosts, we applied peptides at a concentration of 1 µM. In *A. thaliana*, both plant (AtCEP1:hyp4,11) and nematode (MhCEP11:hyp4,11) CEPs reduced the primary root length (Figure 3), while no obvious differences were observed in lateral root numbers (data not shown). The inhibition of primary root length was substantial, exceeding 50% inhibition compared to the control condition. In *M. truncatula,* no effect was seen on root length by MtCEP1D1:hyp4,11 or MhCEP11:hyp4,11 (Figure 4A). We also quantified the number of lateral roots in *M. truncatula* as MtCEP1D1:hyp4,11 has been shown to negatively affect the lateral root number (Imin et la., 2013) In our study, both MtCEP1D1:hyp4,11 and MhCEP11:hyp4,11 reduced the number of lateral roots (Figure 4B). The reduction in lateral root numbers was substantial, with a clear and pronounced effect observed. Compared to the control group without peptide treatment, the reduction amounted to approximately half the number of lateral roots.

### 3.3. M. hapla CEP11 Increases Nitrate Transporter Gene Expression

**Figure 5:**
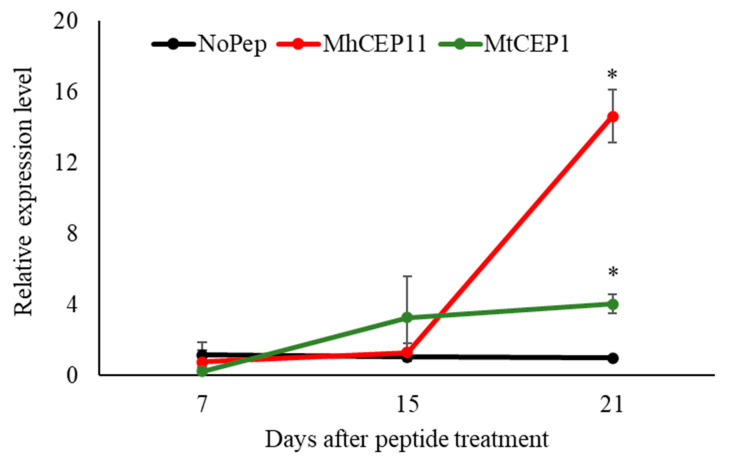
Effects of peptides in nitrate transporter (*NRT2.1*) gene expression temporally. Treatments are compared within each timepoint. Bars on the column represent standard error and bars with asterisk are significantly different (*p* < 0.05) compared to control plants that receive no peptide. The data shown include 3 biological replications.

As plant CEPs are involved in an adaptive response to nitrogen stress conditions [21], we tested for the ability of RKN CEP, MhCEP11:hyp4,11, to upregulate nitrate transporter genes. We used MtCEP1D1:hyp4,11 as a positive control. Peptides for MhCEP11:hyp4,11 and MtCEP1D1:hyp4,11 were chosen for this study as MhCEP11:hyp4,11 is sequentially similar to MtCEP1D1:hyp4,11 with 12 out of 15 amino acid matching and 3 amino acids differing between the sequences [4]. Synthetic peptides to MtCEP1D1:hyp4,11 and MhCEP11:hyp4,11 domains with hydroxyprolines in positions 4 and 11 were exogenously applied to *M. truncatula* seedlings in growth medium. The *NRT2.1* (Medtr4g057890.1) gene expression level was measured at 7 d, 15 d, and 21 d post treatment in peptides. At 7 d and 15 d, there was no significant upregulation of *NRT2.1* expression (Figure 5). However, at 21 d, we observed a notable upregulation (*p* < 0.05, n = 3) of *NRT2.1* expression. Both plant-derived and nematode-derived peptides resulted in a significant upregulation of *NRT2.1* expression, with fold increases of 4 and 14, respectively, compared to plants without peptide treatment. Interestingly, the response to nematode peptides was more pronounced, surpassing the upregulation induced by plant CEP at 21 days when compared to the control group without peptide treatment.

### 3.4. RKN CEP Increases Nitrogen Uptake and Assimilation in M. hapla Feeding Sites

**Figure 6:**
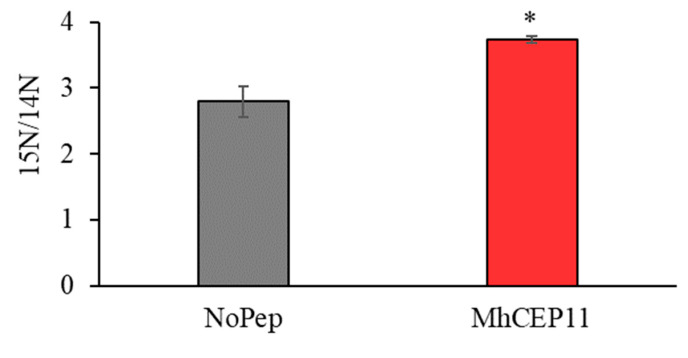
Stable isotope enrichment in *M. hapla* feeding sites at 21 days, as quantified by abundance ratio of 15N/14N with or without exogenous MhCEP11:hyp4,11. Bars on the column represent standard error and bars with asterisk are significantly different (*p* < 0.05) compared to control plants that receive no peptide. The data shown include 3 biological replications.

**Figure 7:**
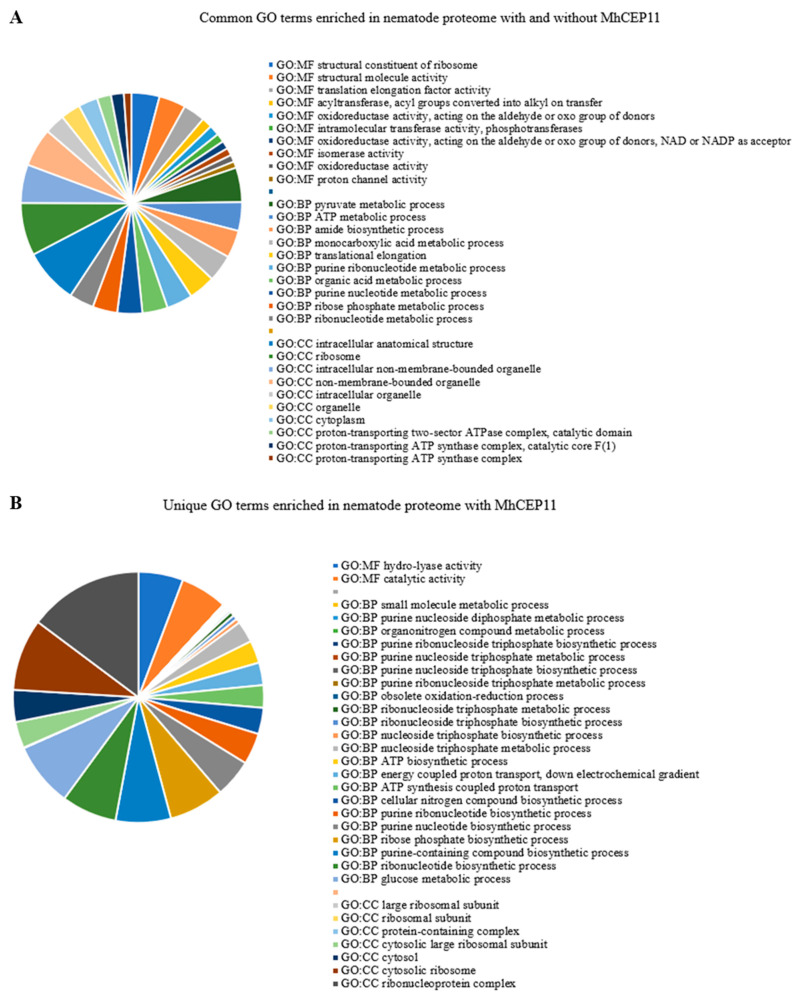
Metabolic processes enriched in *M. hapla* proteome with MhCEP11:hyp4,11. (**A**) Processes enriched in both treatments when plants were supplied with exogenous MhCEP11:hyp4,11 as well as with no peptides, and (**B**) GO terms unique to MhCEP11:hyp4,11 treatment.

CEP plays an important role in the adaptive response to nitrogen stress condition. Specifically, one of the nitrogen pathways regulated by CEP is that of nitrogen transporter genes [21]. We also observed MhCEP11:hyp4,11 involved in nitrogen pathways in plants, as shown by upregulation of the NRT 2.1 gene in the plant at 21 days (Figure 5). We hypothesized that nematode-encoded CEP is involved in commandeering nitrogen allocation pathways in parasitized plants, redirecting nitrogen to the feeding sites or nematode assimilation. To elucidate the possible role of MhCEP11:hyp4,11 in redirecting nitrogen to the feeding site, we labeled plants with the stable isotope N15 and infected them with *Meloidogyne hapla*. Using high-throughput mass spectrometry, we quantified how much plant nitrogen is being assimilated by the nematode. Abundance ratios (15N/14N) quantify stable isotope enrichment in the system. We found that MhCEP11:hyp4,11 increased the nematode assimilation of 15 N at 21 days post inoculation as shown by the higher 15N/14N ratio (Figure 6). To elucidate the metabolic processes involved, we assigned all labeled proteins to enrichment analysis. The average number of proteins identified in samples with MhCEP11:hyp4,11 was 209 and 162 within the no-peptide control; there was no significant difference in the number of proteins identified. Enrichment analysis revealed a higher number of significantly enriched GO terms in the nematode proteome in the presence of exogenous MhCEP11:hyp4,11. There were 61 common GO terms significantly enriched in the nematode proteome with and without MhCEP11:hyp4,11. However, in the presence of MhCEP11:hyp4,11, there were 31 more GO terms significantly enriched, indicating an increase in the number of metabolic pathways and physiological processes occurring when nitrogen assimilation is increased (Figure 7A). Of the 31 unique GO terms, 22 GO terms that are significantly enriched belong to the biological processes including the organonitrogen compound metabolic process and cellular nitrogen compound biosynthetic process. Another seven GP terms that are enriched are involved in molecular and cellular functions (Figure 7B). For the common GO terms, ten highly enriched terms from each molecular (MF), chemical (CF), and biological (BF) function are shown as Supplemental Figure S1.

## 4. Discussion

C-terminally encoded peptides are common to all vascular land plants and more than 900 CEP genes have been identified, with most studies focused on the model plants *A. thaliana* and *M. trunctula* [8,11,13]. CEP-like peptides are also characterized in *Meloidogyne hapla*, a sedentary endoparasite, and in *Rotylenchus reniformis*, a sedentary semi-endoparasite [4,10]. The occurrence of CEP-like signals encoded in plant parasitic nematodes offers a unique opportunity to study the evolution of plant parasitism and endogenous plant signaling. Plant CEP functions include directing the root architecture and adaptive responses to nitrogen stress conditions [19,21]. *R. reniformis* CEP1 demonstrated similar phenotypes and the potential for nitrogen signaling function in *A. thaliana*. As nitrogen is an essential nutrient for growth and development, upregulation of nitrate transporter genes by plant and *R. reniformis* CEPs could provide a plausible benefit to both the plant and nematode. While the structural biology of *Meloidogyne*-encoded CEP has been defined [4], the functional characterization needed to inform nematode parasitism and endogenous signaling was lacking. Here, we elucidated the role of *Meloidogyne*-encoded CEP-like signals in terms of allocating nitrogen and altering plant root system architecture.

While the presence of CEP has been confirmed in different plant parasitic nematode species, its presence throughout a genus has other implications as to the evolution of these signals and the potential to discriminate parasite biology. Mining the seven available RKN genomes with a query built from conserved CEP domains of *A. thaliana* and *M. thuncatula* revealed a total of 61 CEP-like genes. The total numbers of CEP-like genes vary between RKN genomes and do not have a correlation with genome size (Figure 1A). These findings indicate that the presence of CEP-like domains is a common feature among RKN species, with slight variations in the number of peptides observed. The diversity in the number of CEPs suggests potential functional adaptations or specific roles for these peptides within different RKN species The absence of a correlation between genome size and the number of CEPs further highlights the independent evolutionary processes shaping the CEP repertoire in RKN species. Simple sequence analytics in the form of a logo plot showed an expected high sequence similarity of the combined RKN CEP domain with the individual plant CEP domain (Figure 1B), supporting previous reports of plant and nematode sequence similarity [10]. A phylogenetic tree of plant CEPs and nematode CEP-like domains does not show distinct clustering with plant species (Figure 2); indeed, nematode CEPs are dispersed, indicating a similarity and relatedness between specific RKN and plant CEPs. This supports the hypothesis that RKN CEPs are functional mimics of plant CEP. While many plant CEP functions have been characterized, there are likely more; some that are even species-specific and have corresponding functional mimics encoded in the RKN genomes sustain their biotrophic interaction with a wide and diverse host range. 

Altering plant development and nutrient use is not only reserved for the CEP family of peptides. For example, genetic analysis in legumes has revealed a central role for CLE in the shoot-root circuit that regulates the number of nitrogen-fixing nodules able to be induced by rhizobacteria [36,37]. Furthermore, plant parasitic nematodes are also known to encode CLE-like peptides [38] and have impacts on parasitism such that *Lotus japonicas* carrying hyper-nodulating alleles of *har-1*, which is an ortholog of the canonical Arabidopsis CLE receptor *clv1*, exhibits a significantly higher infection with nematodes (Lohar and Bird, 2003) compared to wild-type plants. Beyond legumes, natural sequence polymorphisms at an orthologous locus in tomato correlate with RKN host range [39]. Such variations in peptide sequences between families with overlapping roles in plant development and nitrogen assimilation seem to indicate a high degree of functional plasticity derived from amino acid content and primary structure. Thus, even small variations, such as those between RKN and plant-encoded CEP (or CLE) peptides, can have significant or, equally, insignificant impacts on function and necessitate biological experimentation. 

Previous work on obtaining the tertiary structure of *M. hapla* and *M. truncatula* CEPs have shown the sequential and structural similarity of MhCEP11:hyp4,11 and MtCEP1D1:hyp4,11 [4] with 12 out of 15 amino acids matching and 3 amino acids differing between these two sequences. However, studies have shown that even a small difference in the amino acid sequence can lead to significant differences in biological activity and function [6,40]. Therefore, we choose to focus our work on these two peptides to better describe their functional roles in root architecture and nitrogen signaling. CEP plays a role in manipulating the integral components of root architecture including primary root length and number of lateral roots [11,12,13]. MhCEP11:hyp4,11 and AtCEP1:hyp4,11 produced a similar root phenotype in *A. thaliana* with reduced primary root length, while MhCEP11:hyp4,11 and MtCEP1D1:hyp4,11 in *M. truncatula* had no effect on root length. CEP-inhibited root proliferation through limiting the rate of cell division has been demonstrated in *A. thaliana* [11]. Both MhCEP11:hyp4,11 and MtCEP1D1:hyp4,11 reduced the number of lateral roots produced in *M. truncatula.* Under nitrogen stress conditions, *M. truncatula* is prone to nodulation [9,41] like other legumes. This reduction in the number of lateral roots may be a compensating phenotype for increased nodule formations [42]. The RKN CEP, MhCEP11:hyp4,11, reduced the lateral root number and increased the expression of *NRT2.1* in *Medicago*, suggesting that the cortical hyperplasia seen surrounding RKN-induced ‘giant cells’ could be partly explained by CEP and explain other similarities between root-knot galls and nitrogen-fixing nodules [43].

Our temporal analysis of nitrate transporter gene expression showed that exogenous application of synthetic MhCEP11:hyp4,11 and MtCEP1D1:hyp4,11 upregulates *NRT2.1* at 21 d post treatment under nitrogen starving conditions (Figure 5). The upregulation of *NRT2.1* in *A. thaliana* through exogenous CEP application has been demonstrated in another sedentary parasite [10]. We did not observe the upregulation of nitrate transporter genes until two weeks post treatment in peptides. There are several reasons for this discrepancy, noting differences in biological assays and timing, as well as inherent chemistries of the peptides dictating different plant uptake rates. Additionally, there could be differences between individual CEP functions related to the differences in parasitic nematode biology. Further, as plants grow older, the demand for nitrogen could have caused enhanced the expression of *NRT2.1* as an adaptive response to nitrogen stress conditions that could benefit both plant and nematodes. 

As CEPs are involved in the expression of nitrate transporter genes, there is a possibility that nematode-encoded CEPs are involved in nitrogen allocation pathways in infected plants, redirecting nitrogen to feeding sites to support parasite growth and reproduction. We utilized stable isotope labeling coupled with mass spectrometry to quantify how much plant nitrogen is being assimilated by the nematode with and without the application of exogenous nematode MhCEP11:hyp4,11. Stable isotope labeling involves the incorporation of a scarce stable isotope into the proteome of an organism [44]. This ‘labeling’ allows for accurate and sensitive comparative proteomic analyses as the added mass is readily identified through spectrometry. This approach has been used to demonstrate the function of MtCEP1D1:hyp4,11 of increasing the uptake of macronutrients, including nitrogen [24]. Importantly, sequentially distinct CEPs have distinct roles in nutrient uptake and were always able to function across plant species. Here, we demonstrate that MhCEP11:hyp4,11 increases the assimilation of nematode nitrogen, probably by redirecting plant nutrients to feeding sites and enhancing several metabolic processes. There were 31 unique metabolic processes increased in nematodes feeding sites with MhCEP11:hyp4,11 application. Most of the enriched processes involved purine metabolic and nitrogen compound biosynthetic processes. Purine synthesis has been shown to play an important role in the primary metabolism of nitrogen, including the transport and storage of organic nitrogen in some legume crops [45]. The cellular nitrogen compound biosynthetic process involves the chemical reaction and pathways, resulting in the formation of organic and inorganic nitrogenous compounds (based on gene ontology). Together, these data indicate the involvement of RKN CEP in nitrogen signaling (Figure 7). 

Identifying the function of RKN CEP in expressing nitrate transporter gene and root phenotypes indicates its importance in nitrogen signaling. The increased enrichment ratio (15N/14N) in nematode feeding sites also indicates that nematode peptide MhCEP11:hyp4,11 is involved in directing plant nitrogen to their feeding site. That suggests a broader role of CEP in plant biology and probably benefits both nematodes and plants. The wide host range of RKN, coupled with the ability to alter plant nutrient uptake and allocation, makes this system an ideal model to increase nutrient use efficiency. 

## Data Availability

All data supporting the findings of this study are available within the paper and within its supplementary materials published online.

## Supplementary Materials

The following supporting information can be downloaded at https://www.mdpi.com/article/10.3390/life13102020/s1, Table S1: Sequences of CEP-like peptides discovered in RKN genomes.

## References

[1] Rutter W.B., Franco J., Gleason C. (2022). Rooting Out the Mechanisms of Root-Knot Nematode-Plant Interactions. Annu. Rev. Phytopathol..

[2] Bartlem D.G., Jones M.G.K., Hammes U.Z. (2014). Vascularization and Nutrient Delivery at Root-Knot Nematode Feeding Sites in Host Roots. J. Exp. Bot..

[3] Hewezi T. (2015). Cellular Signaling Pathways and Posttranslational Modifications Mediated by Nematode Effector Proteins. Plant Physiol..

[4] Bobay B.G., Digennaro P., Scholl E., Imin N., Djordjevic M.A., McK Bird D. (2013). Solution NMR Studies of the Plant Peptide Hormone CEP Inform Function. FEBS Lett..

[5] Mitchum M.G., Wang X., Wang J., Davis E.L. (2012). Role of Nematode Peptides and Other Small Molecules in Plant Parasitism. Annu. Rev. Phytopathol..

[6] Hirakawa Y., Sawa S. (2019). Diverse Function of Plant Peptide Hormones in Local Signaling and Development. Curr. Opin. Plant Biol..

[7] Furumizu C., Sawa S. (2021). The RGF/GLV/CLEL Family of Short Peptides Evolved Through Lineage-Specific Losses and Diversification and Yet Conserves Its Signaling Role Between Vascular Plants and Bryophytes. Front. Plant Sci..

[8] Ogilvie H.A., Imin N., Djordjevic M.A. (2014). Diversification of the C-TERMINALLY ENCODED PEPTIDE (CEP) Gene Family in Angiosperms, and Evolution of Plant-Family Specific CEP Genes. BMC Genom..

[9] Ohyama K., Ogawa M., Matsubayashi Y. (2008). Identification of a Biologically Active, Small, Secreted Peptide in Arabidopsis by in Silico Gene Screening, Followed by LC-MS-Based Structure Analysis. Plant J..

[10] Eves-Van Den Akker S., Lilley C.J., Yusup H.B., Jones J.T., Urwin P.E. (2016). Functional C-TERMINALLY ENCODED PEPTIDE (CEP) Plant Hormone Domains Evolved de Novo in the Plant Parasite Rotylenchulus Reniformis. Mol, Plant Pathol..

[11] Delay C., Imin N., Djordjevic M.A. (2013). CEP Genes Regulate Root and Shoot Development in Response to Environmental Cues and Are Specific to Seed Plants. J. Exp. Bot..

[12] Mohd-Radzman N.A., Binos S., Truong T.T., Imin N., Mariani M., Djordjevic M.A. (2015). Novel MtCEP1 Peptides Produced in Vivo Differentially Regulate Root Development in Medicago Truncatula. J. Exp. Bot..

[13] Imin N., Mohd-Radzman N.A., Ogilvie H.A., Djordjevic M.A. (2013). The Peptide-Encoding CEP1 Gene Modulates Lateral Root and Nodule Numbers in Medicago Truncatula. J. Exp. Bot..

[14] Chapman K., Ivanovici A., Taleski M., Sturrock C.J., Ng J.L.P., Mohd-Radzman N.A., Frugier F., Bennett M.J., Mathesius U., Djordjevic M.A. (2020). CEP Receptor Signalling Controls Root System Architecture in Arabidopsis and Medicago. New Phytol..

[15] Amano Y., Tsubouchi H., Shinohara H., Ogawa M., Matsubayashi Y. (2007). Tyrosine-Sulfated Glycopeptide Involved in Cellular Proliferation and Expansion in Arabidopsis. Proc. Natl. Acad. Sci. USA.

[16] Chen Y.F., Matsubayashi Y., Sakagami Y. (2000). Peptide Growth Factor Phytosulfokine-Alpha Contributes to the Pollen Population Effect. Planta.

[17] Ito Y., Nakanomyo I., Motose H., Iwamoto K., Sawa S., Dohmae N., Fukuda H. (2006). Dodeca-CLE Peptides as Suppressors of Plant Stem Cell Differentiation. Science.

[18] Matsubayashi Y. (2014). Posttranslationally Modified Small-Peptide Signals in Plants. Annu. Rev. Plant Biol..

[19] Delay C., Chapman K., Taleski M., Wang Y., Tyagi S., Xiong Y., Imin N., Djordjevic M.A. (2019). CEP3 Levels Affect Starvation-Related Growth Responses of the Primary Root. J. Exp. Bot..

[20] Gansel X., Muños S., Tillard P., Gojon A. (2001). Differential Regulation of the NO3- and NH4+ Transporter Genes AtNrt2.1 and AtAmt1.1 in Arabidopsis: Relation with Long-Distance and Local Controls by N Status of the Plant. Plant J..

[21] Tabata R., Sumida K., Yoshii T., Ohyama K., Shinohara H., Matsubayashi Y. (2014). Perception of Root-Derived Peptides by Shoot LRR-RKs Mediates Systemic N-Demand Signaling. Science.

[22] Ohkubo Y., Tanaka M., Tabata R., Ogawa-Ohnishi M., Matsubayashi Y. (2017). Shoot-to-Root Mobile Polypeptides Involved in Systemic Regulation of Nitrogen Acquisition. Nat. Plants.

[23] Taleski M., Imin N., Djordjevic M.A. (2018). CEP Peptide Hormones: Key Players in Orchestrating Nitrogen-Demand Signalling, Root Nodulation, and Lateral Root Development. J. Exp. Bot..

[24] Roy S., Griffiths M., Torres-Jerez I., Sanchez B., Antonelli E., Jain D., Krom N., Zhang S., York L.M., Scheible W.R. (2022). Application of Synthetic Peptide CEP1 Increases Nutrient Uptake Rates Along Plant Roots. Front. Plant Sci..

[25] Mathesius U. (2003). Conservation and Divergence of Signalling Pathways between Roots and Soil Microbes—The Rhizobium-Legume Symbiosis Compared to the Development of Lateral Roots, Mycorrhizal Interactions and Nematode-Induced Galls. Plant Soil.

[26] Bird D.M.K. (2004). Signaling between Nematodes and Plants. Curr. Opin. Plant Biol..

[27] Costa S.R., Pin Ng J.L., Mathesius U. (2021). Interaction of Symbiotic Rhizobia and Parasitic Root-Knot Nematodes in Legume Roots: From Molecular Regulation to Field Application. Mol. Plant Microbe Interact..

[28] Mitchum M.G., Wang X., Davis E.L. (2008). Diverse and Conserved Roles of CLE Peptides. Curr. Opin. Plant Biol..

[29] Rice P., Longden L., Bleasby A. (2000). EMBOSS: The European Molecular Biology Open Software Suite. Trends Genet..

[30] Schwarz R., Dayhoff M., Dayhoff M. (1979). Matrices for Detecting Distant Relationships. Atlas of Protein Sequences.

[31] Kumar S., Stecher G., Li M., Knyaz C., Tamura K. (2018). MEGA X: Molecular Evolutionary Genetics Analysis across Computing Platforms. Mol. Biol. Evol..

[32] Fahraeus G. (1957). The Infection of Clover Root Hairs by Nodule Bacteria Studied by a Simple Glass Slide Technique. J. Gen. Microbiol..

[33] Asai S., Rallapalli G., Piquerez S.J.M., Caillaud M.C., Furzer O.J., Ishaque N., Wirthmueller L., Fabro G., Shirasu K., Jones J.D.G. (2014). Expression Profiling during Arabidopsis/Downy Mildew Interaction Reveals a Highly-Expressed Effector That Attenuates Responses to Salicylic Acid. PLoS Pathog..

[34] Holmes P., Goffard N., Weiller G.F., Rolfe B.G., Imin N. (2008). Transcriptional Profiling of Medicago Truncatula Meristematic Root Cells. BMC Plant Biol.

[35] Raudvere U., Kolberg L., Kuzmin I., Arak T., Adler P., Peterson H., Vilo J. (2019). G:Profiler: A Web Server for Functional Enrichment Analysis and Conversions of Gene Lists (2019 Update). Nucleic Acids Res..

[36] Okamoto S., Ohnishi E., Sato S., Takahashi H., Nakazono M., Tabata S., Kawaguchi M. (2009). Nod Factor/Nitrate-Induced CLE Genes That Drive HAR1-Mediated Systemic Regulation of Nodulation. Plant Cell Physiol..

[37] Mortier V., den Herder G., Whitford R., van de Velde W., Rombauts S., D’haeseleer K., Holsters M., Goormachtig S. (2010). CLE Peptides Control Medicago Truncatula Nodulation Locally and Systemically. Plant Physiol..

[38] Frei dit Frey N., Favery B. (2021). Plant-Parasitic Nematode Secreted Peptides Hijack a Plant Secretory Pathway. New Phytol..

[39] Ejima C., Uwatoko T., Thi Ngan B., Honda H., Shimizu N., Kiyohara S., Hamasaki R., Sawa S. (2011). SNPs of CLAVATA Receptors in Tomato, in the Context of Root-Knot Nematode Infection. Nematol. Res..

[40] Shi Y., Sun S., Zhang Y., He Y., Du M., ÓReilly A.O., Wu S., Yang Y., Wu Y. (2022). Single Amino Acid Variations Drive Functional Divergence of Cytochrome P450s in Helicoverpa Species. Insect Biochem. Mol. Biol..

[41] Jiang Y., MacLean D.E., Perry G.E., Marsolais F., Hill B., Pauls K.P. (2020). Evaluation of Beneficial and Inhibitory Effects of Nitrate on Nodulation and Nitrogen Fixation in Common Bean (Phaseolus Vulgaris). Legume Sci..

[42] Mergaert P., Kereszt A., Kondorosi E. (2020). Gene Expression in Nitrogen-Fixing Symbiotic Nodule Cells in Medicago Truncatula and Other Nodulating Plants. Plant Cell.

[43] Weerasinghe R.R., Bird D.M.K., Allen N.S. (2005). Root-Knot Nematodes and Bacterial Nod Factors Elicit Common Signal Transduction Events in Lotus Japonicus. Proc. Natl. Acad. Sci. USA.

[44] Wu C.C., MacCoss M.J., Howell K.E., Matthews D.E., Yates J.R. (2004). Metabolic Labeling of Mammalian Organisms with Stable Isotopes for Quantitative Proteomic Analysis. Anal. Chem..

[45] Zrenner R., Stitt M., Sonnewald U., Boldt R. (2006). Pyrimidine and Purine Biosynthesis and Degradation in Plants. Annu. Rev. Plant Biol..

