# Supplementary material for: Root-Knot-Nematode-Encoded CEPs Increase Nitrogen Assimilation
Source: Life (Basel). 2023 Oct 7;13(10):2020. doi: 10.3390/life13102020 (PMC10608282; doi:10.3390/life13102020)
Supplement: Supplementary file 1 [file life-13-02020-s001.zip › life-2564659-supplementary.pdf]

Supplemental Table S1. Sequences of CEP-like peptides discovered in RKN genomes

RKN Species	CEP Names	CEP Sequences
M. hapla (PRJNA29083)	MhCEP1	DFRPTNPGHSPGIGH
	MhCEP2	AFRPTAPGHSPGVGH
	MhCEP3	GYQPTNPGHSPGIGH
	MhCEP4	FKTVPGQSSPGVGH
	MhCEP5	VIKPACIGNSPGVGH
	MhCEP6	AFRPTNPGPSPAIGH
	MhCEP7	GFRPTNPGNSPGAGH
	MhCEP8	TFRPTAPGHSPGIGN
	MhCEP9	PFCTGPGHSPGVGH
	MhCEP10	PFCTGSGHSPGVGH
	MhCEP11	AFRPTAPGHSPGVGH
	MhCEP12	PFHTGTGRSPGAGH
M. graminicola (PRJNA411966)	MgCEP1	FRVLKPGGSPVGLG
	MgCEP2	HTPGVG
M. incognita (PRJEB8714)	MiCEP1	RPTEPGHSPGAGH
	MiCEP2	FRPTNPGPSPGVGH
	MiCEP3	PTGPGHSPGVGN
	MiCEP4	RATEPGHSPGAGH
	MiCEP5	DTRPTEPGHSPGAGH
	MiCEP6	RPTQPGHSPGVGN
	MiCEP7	TEPGHSPGAGH
	MiCEP8	RPTNPGHSPGAGH
	MiCEP9	DVHPNNPGHSPGIGH
	MiCEP10	RPTQPGHSPGVGN
	MiCEP11	VHPNNPGHSPGIGH
M. arenaria (PRJNA438575)	MaCEP1	FRSTCPASSAAA
	MaCEP2	QPIHSPSYSH
	MaCEP3	FNPNNLPGNCP
	MaCEP4	FNPNNLPGNCP
	MaCEP5	SFRSTCPASSAAA
	MaCEP6	RPTFPG
	MaCEP7	SFRSTCPASSAAA
M. enterolobii (PRJNA340324)	MeCEP1	FRPTVPGHSPGIGH
	MeCEP2	FQPTSPGHSPGIGH
	MeCEP3	IKPTNPGHSPGGGH
	MeCEP4	DKRATNPGHSPGVGN
	MeCEP5	DTSKPTAPGHSPGIGH
	MeCEP6	FQPTAPGHSPGIGH
	MeCEP7	DYRQTHPGNSPGIGH
	MeCEP8	DYRETHPGNSPGIGH
	MeCEP9	DYRQTHPGNSPGIGH
	MeCEP10	DYRPTEPGHSPGVGH
	MeCEP11	DKRPTAPGHSPGIGH
	MeCEP12	DKRQTNPGNSPGVGN
	MeCEP13	DKRATNPGHSPGVGN
	MeCEP14	DKRATNPGHSPGVGN
M. floridensis (PRJNA340324)	MfCEP1	DVHPNNPGHSPGIGH
	MfCEP2	DTRPTEPGHSPGAGH
	MfCEP3	RATEPGHSPGAGH
	MfCEP4	RATEPGHSPGAGH
	MfCEP5	DFRPTNPGHSPGVGH
	MfCEP6	RPTQPGHSPGVG
	MfCEP7	RPTNPGHSPGAGH

	MfCEP8	RPTNPGHSPGAGH
M. javanica (PRJNA340324)	MjCEP1	DVHPNNPGHSPGIGH
	MjCEP2	DTRPTEPGHSPGAGH
	MjCEP3	RPTNPGHSPGAGH
	MjCEP4	DFRPTYPGHSPGVGH
	MjCEP5	RPTGPGHSPGVGN
	MjCEP6	RATEPGHSPGAGH
	MjCEP7	DVHPNNPGHSPGVGH
	MjCEP8	DFRPTNPGHSPGVGH
	MjCEP9	RSTGPGHSPGVGNH
	MjCEP10	RPTEPGHSPGAGH
	MjCEP11	FRPTNPGPSPGVGH
	MjCEP12	DFRETNPGKSPRIGH
	MjCEP13	TAPGHSPGIGN
	MjCEP14	DVHPNNPGHSPGIGH
